# Gender differences in the association between adverse childhood experiences and impulsivity in Saudi Arabian adults: A cross-sectional study

**DOI:** 10.1371/journal.pone.0341789

**Published:** 2026-05-04

**Authors:** Saleh A. Alghamdi

**Affiliations:** Department of Psychiatry, College of Medicine, Imam Mohammad Ibn Saud Islamic University (IMSIU), Riyadh, Saudi Arabia; University of Toronto, CANADA

## Abstract

**Background:**

Adverse childhood experiences (ACEs) are linked to impulsivity, a transdiagnostic risk factor for psychopathology. However, gender differences in ACE-impulsivity pathways remain unclear, particularly in Middle Eastern populations, and prior research has often conflated differential exposure with differential vulnerability while lacking rigorous multiple testing corrections.

**Objective:**

This study examined gender differences in ACE exposure, multidimensional impulsivity, and ACE-impulsivity associations in Saudi Arabian adults, distinguishing between differential exposure and differential vulnerability hypotheses using rigorous statistical methods.

**Methods:**

A nationwide cross-sectional online survey recruited 379 Saudi adults (222 female, 157 male; majority aged 18–25 years [55.7%]). Participants completed the Arabic 10-item ACE questionnaire and Short UPPS-P Impulsive Behavior Scale (five dimensions: Negative Urgency, Positive Urgency, Sensation Seeking, Lack of Premeditation, Lack of Perseverance). Analyses employed Benjamini-Hochberg false discovery rate (FDR) correction (q = .05) across test families, bootstrap confidence intervals (5,000 iterations) for all effect sizes, and hierarchical regression models testing Gender × ACE interactions with demographic covariates.

**Results:**

Females reported significantly higher ACE exposure (M = 2.24 vs. 1.75, p = .005), concentrated in emotional neglect (35.1% vs. 17.8%, FDR-significant) and household mental illness (29.3% vs. 16.6%, FDR-significant). No impulsivity dimension showed significant gender differences after FDR correction. Gender-stratified analyses revealed significant ACE correlations for four dimensions in females (ρ = 0.142–0.262) but only Negative Urgency in males (ρ = 0.222). However, bootstrap tests of cross-gender correlation differences were all non-significant (all Δρ 95% CIs included zero). Covariate-adjusted Gender × ACE interactions were uniformly null (all p > .16, ΔR² < 0.005).

**Conclusions:**

Gender differences in ACE-impulsivity associations reflect differential exposure (higher female ACE burden) rather than differential vulnerability (different per-ACE impact). Findings support universal trauma-informed interventions with ACE-type-specific modifications rather than fundamentally gender-differentiated approaches. Results highlight the importance of rigorous statistical controls and distinction between exposure and vulnerability in ACE research.

## Introduction

### Adverse childhood experiences: Prevalence and consequences

Adverse childhood experiences (ACEs) encompass a range of potentially traumatic events occurring before age 18, including abuse, neglect, and household dysfunction [1]. Since the landmark ACE Study, substantial research has documented the high prevalence and long-term consequences of childhood adversity. In the United States, approximately 61% of adults report at least one ACE, and nearly 16% report four or more ACEs [2]. Internationally, prevalence estimates vary considerably by region, with particularly limited data from Middle Eastern countries [3,4].

The consequences of ACEs extend across the lifespan, affecting physical health, mental health, and behavioral outcomes [5]. Meta-analytic evidence demonstrates robust associations between ACEs and depression, anxiety, substance use disorders, and suicidal behavior [6,7]. Beyond clinical diagnoses, ACEs influence dimensional personality traits and self-regulatory capacities that underlie psychopathology risk [8]. Among these, impulsivity has emerged as a key transdiagnostic pathway linking childhood adversity to maladaptive outcomes [9].

### Impulsivity as a multidimensional construct

Impulsivity is increasingly recognized as a multifaceted construct rather than a unitary trait [10]. The UPPS-P model, one of the most empirically supported frameworks, delineates five distinct dimensions: Negative Urgency (rash action during negative affect), Positive Urgency (rash action during positive affect), Sensation Seeking (pursuit of novel and thrilling experiences), Lack of Premeditation (acting without forethought), and Lack of Perseverance (inability to maintain focus on tasks) [11,12]. These dimensions demonstrate differential associations with psychopathology; for example, urgency facets predict emotion-driven behaviors such as binge eating and non-suicidal self-injury, whereas sensation seeking relates more strongly to substance experimentation and risky sexual behavior [13,14].

Neurobiologically, impulsivity reflects deficits in prefrontal regulatory systems, particularly those involving executive function and emotion regulation [15]. Childhood adversity may compromise these regulatory capacities through multiple mechanisms, including altered stress response systems, disrupted attachment relationships, and structural brain changes in regions critical for impulse control [16,17]. Longitudinal studies suggest that early adversity predicts elevated impulsivity in adolescence and adulthood, which in turn mediates risk for substance use, aggression, and other externalizing problems [18,19].

### Gender differences in ACE exposure

Gender differences in ACE exposure are well-documented, though patterns vary across ACE types and cultural contexts [20,21]. In Western samples, females typically report higher rates of sexual abuse, whereas males report higher rates of physical abuse and neglect [22,23]. However, these patterns may not generalize to non-Western contexts with different gender role norms and family structures. For emotional abuse and neglect, findings are mixed, with some studies reporting higher female prevalence and others finding no significant differences [24,25].

Beyond prevalence differences, accumulating evidence suggests gender may moderate the impact of ACEs on developmental outcomes—a phenomenon termed “differential vulnerability” [26]. Some research indicates females show stronger associations between childhood adversity and internalizing psychopathology, whereas males show stronger associations with externalizing problems, potentially reflecting gender-specific coping styles and socialization processes [27,28]. However, other studies find comparable ACE effects across genders, supporting “differential exposure” rather than differential vulnerability hypotheses [29,30].

### Gender differences in impulsivity

Research on gender differences in impulsivity has yielded complex and sometimes contradictory findings. Early meta-analyses suggested males exhibit higher trait impulsivity overall, particularly on sensation seeking dimensions [31]. However, more recent work using multidimensional frameworks reveals a nuanced pattern: males consistently score higher on sensation seeking, whereas females often score higher on urgency dimensions, particularly negative urgency [32,33]. This pattern aligns with broader gender differences in emotion regulation [34] and suggests females may be especially vulnerable to emotion-driven impulsive behaviors.

Importantly, most research on gender differences in impulsivity has been conducted in Western, educated, industrialized, rich, and democratic (WEIRD) societies, limiting generalizability to other cultural contexts [35]. Cultural factors influence both the expression and perception of impulsive behavior, with potential implications for gender differences [36]. In collectivistic cultures, where emotional restraint and conformity to social norms may be particularly emphasized for women, gender differences in self-reported impulsivity may manifest differently than in individualistic Western contexts.

### ACE–impulsivity associations: Gender as a moderator

A growing body of research examines associations between ACEs and impulsivity, generally finding positive correlations [9,37]. However, relatively few studies have explicitly tested whether these associations differ by gender. Among studies that have examined gender as a moderator, findings are inconsistent. Some report stronger ACE-impulsivity associations in females, particularly for emotion-driven impulsivity dimensions, consistent with differential vulnerability to emotional sequelae of trauma [38]. Others find no significant moderation by gender, suggesting comparable per-ACE impacts across genders despite different exposure patterns [39].

These inconsistencies may reflect methodological limitations in the existing literature. Many studies treat impulsivity as unidimensional, potentially obscuring dimension-specific gender effects. Additionally, most studies fail to distinguish between differential exposure (group differences in ACE prevalence) and differential vulnerability (group differences in the strength of ACE-outcome associations), conflating these conceptually distinct phenomena [40]. Failure to apply rigorous corrections for multiple testing, use of inappropriate parametric methods for ordinal data, and omission of relevant demographic covariates further complicate interpretation of gender moderation effects.

### Middle Eastern context and cultural considerations

Research on ACEs and their psychological consequences in Middle Eastern populations remains remarkably limited, despite the region comprising over 400 million people across diverse cultural contexts. Available evidence suggests ACE prevalence in the Middle East may be substantial, though reporting rates are likely suppressed by cultural stigma surrounding family problems and mental health [3]. In Saudi Arabia specifically, the few existing studies estimate that 40–50% of adults have experienced at least one ACE, with particularly high rates of emotional abuse and neglect [41,42].

Cultural factors unique to Saudi society may influence both ACE exposure patterns and their psychological consequences. Saudi Arabia’s patriarchal family structure, with clearly delineated gender roles and expectations, may contribute to gender-specific vulnerabilities [43]. Women in Saudi society have historically faced restrictions on autonomy, education, and social participation, though recent reforms have begun to address some of these limitations as part of Vision 2030 initiatives [44]. These sociocultural factors may influence not only ACE exposure—particularly emotional neglect and household dysfunction—but also pathways from adversity to outcomes such as impulsivity.

For males, cultural expectations of emotional stoicism and the stigma surrounding mental health problems may suppress disclosure of certain ACE types, particularly emotional and sexual abuse [45]. Conversely, females may face greater family control and psychological pressure, potentially elevating risk for emotional abuse and neglect while simultaneously restricting behavioral expressions of impulsivity due to social consequences. These gendered cultural dynamics necessitate culturally informed research examining whether Western models of ACE-impulsivity associations generalize to Saudi and other Middle Eastern contexts.

### The present study

Despite substantial research on ACEs and impulsivity in Western populations, critical gaps remain. First, no published studies have examined gender differences in ACE-impulsivity associations using multidimensional impulsivity frameworks in Middle Eastern populations. Second, existing research often conflates differential exposure and differential vulnerability, failing to rigorously test moderation while controlling for demographic confounders. Third, many prior studies lack appropriate corrections for multiple testing, potentially inflating Type I error rates and overstating gender differences.

The present study addressed these gaps by examining gender differences in ACE exposure, impulsivity, and ACE-impulsivity associations in a nationwide Saudi Arabian adult sample. We employed a multidimensional assessment of impulsivity using the Short UPPS-P Impulsive Behavior Scale [46], allowing examination of dimension-specific effects. Critically, we distinguished between differential exposure (Do males and females differ in ACE prevalence?) and differential vulnerability (Does gender moderate the strength of ACE-impulsivity associations?) using formal moderation analyses with demographic covariate adjustment. All analyses incorporated rigorous statistical corrections including Benjamini-Hochberg false discovery rate (FDR) control for multiple comparisons and bootstrap confidence intervals for effect size estimation.

We hypothesized that: (1) females would report higher ACE exposure overall, particularly for emotion-laden ACE types such as emotional abuse and emotional neglect; (2) gender differences in mean impulsivity levels would be dimension-specific, with potentially higher urgency in females and higher sensation seeking in males; (3) ACEs would positively correlate with impulsivity dimensions in both genders, with potentially stronger associations in females for urgency dimensions; and (4) formal moderation tests would clarify whether apparent gender differences reflect differential exposure versus differential vulnerability. Given the exploratory nature of Middle Eastern ACE research and the cultural specificity of gender dynamics in Saudi Arabia, we remained open to patterns that might diverge from Western findings.

## Materials and methods

### Participants and procedure

This cross-sectional study was approved by the Institutional Review Board of Imam Mohammad Ibn Saud Islamic University (Project #717/2024, Session 73). Participants were recruited via social media and university email lists from December 14, 2024 to July 26, 2025 using convenience sampling. Eligibility criteria included: Saudi nationality/long-term residency, age ≥ 18 years, and Arabic literacy. All participants provided electronic informed consent.

The final sample comprised 379 adults (222 female [58.6%], 157 male [41.4%]) with complete data. Mean age category was 18–25 years (55.7%), with representation from all five Saudi regions (Central 75.2%, Western 10.3%, Eastern 8.2%, Southern 3.4%, Northern 2.9%). Most participants held bachelor’s degrees (46.7%) and were students (49.6%) or employed (35.9%). Twenty-five percent reported a psychiatric diagnosis, most commonly depression or anxiety.

Sample size was determined using G*Power 3.1.9.7. To detect a small-to-medium interaction effect (f² = 0.04) with 80% power and α = .05, a minimum of 322 participants was required; our sample of 379 exceeded this threshold.

The anonymous online survey (Google Forms) required 15–20 minutes. Forced-response formatting ensured zero missing data on primary variables. IP restrictions prevented duplicate submissions, and attention checks verified data quality.

### Measures

#### Adverse childhood experiences.

The Arabic 10-item ACE questionnaire [1,42] assessed abuse (emotional, physical, sexual), neglect (emotional, physical), and household dysfunction (parental separation, domestic violence, substance abuse, mental illness, incarceration) before age 18. Items were scored dichotomously (0 = no, 1 = yes) and summed (range: 0–10). Internal consistency was acceptable (KR-20 = .72). ACE categories were created following established conventions: 0, 1, 2–3, and 4 + ACEs [2].

#### Impulsivity.

The Arabic Short UPPS-P Impulsive Behavior Scale [46] assessed five dimensions with four items each: Negative Urgency (rash action during negative affect), Positive Urgency (rash action during positive affect), Sensation Seeking (novelty/thrill-seeking), Lack of Premeditation (acting without forethought), and Lack of Perseverance (poor task focus). Items used 4-point Likert scales (1 = *strongly disagree* to 4 = *strongly agree*). Internal consistency was good for four subscales (α:.70−.82) but below threshold for Lack of Premeditation (α = .55), consistent with prior short-form research in non-Western samples [47].

#### Demographics.

Participants reported age, gender, region, education, employment, marital status, and psychiatric diagnosis history.

### Sample characteristics and preliminary analyses

The final sample comprised 379 Saudi adults (222 female, 157 male) with complete data on all primary variables. Table 1 presents demographic characteristics by gender. Females and males differed significantly in regional distribution (χ²(4) = 23.13, p < .001, Cramér’s V = 0.25), employment status (χ²(3) = 16.99, p = .001, V = 0.21), and marital status (χ²(2) = 12.62, p = .002, V = 0.18), but not in age distribution (χ²(4) = 2.48, p = .649) or educational level (χ²(4) = 1.55, p = .670) (Table 1). These demographic differences justified inclusion of covariates in moderation models.

**Table 1:** Sample demographic characteristics by gender.

Variable	Female (n = 222)	Male (n = 157)	Test Statistic	p-value
**Age Category**			χ²(4) = 2.48	.649
18–25 years	128 (57.7%)	83 (52.9%)		
26–35 years	46 (20.7%)	34 (21.7%)		
36–45 years	17 (7.7%)	16 (10.2%)		
46–55 years	21 (9.5%)	15 (9.6%)		
56–65 years	10 (4.5%)	9 (5.7%)		
**Region**			χ²(4) = 23.13	<.001***
Central	181 (81.5%)	104 (66.2%)		
Western	18 (8.1%)	21 (13.4%)		
Eastern	13 (5.9%)	18 (11.5%)		
Southern	6 (2.7%)	7 (4.5%)		
Northern	4 (1.8%)	7 (4.5%)		
**Education Level**			χ²(4) = 1.55	.670
High school	44 (19.8%)	35 (22.3%)		
Diploma	20 (9.0%)	16 (10.2%)		
Bachelor’s	105 (47.3%)	72 (45.9%)		
Master’s	42 (18.9%)	28 (17.8%)		
PhD	11 (5.0%)	6 (3.8%)		
**Employment Status**			χ²(3) = 16.99	.001**
Student	123 (55.4%)	65 (41.4%)		
Employed	68 (30.6%)	68 (43.3%)		
Unemployed	26 (11.7%)	11 (7.0%)		
Retired	5 (2.3%)	13 (8.3%)		
**Marital Status**			χ²(2) = 12.62	.002**
Single	166 (74.8%)	98 (62.4%)		
Married	49 (22.1%)	52 (33.1%)		
Divorced	7 (3.2%)	7 (4.5%)		
**Psychiatric Diagnosis**	62 (27.9%)	34 (21.7%)	χ²(1) = 2.10	.147

*Note.* ** p < .01. *** p < .001.

### Gender differences in ACE exposure

Females reported significantly higher ACE total scores (M = 2.24, SD = 2.20, Mdn = 2.0) compared to males (M = 1.75, SD = 2.25, Mdn = 1.0), Mann-Whitney U = 20,270.5, p = .005, rank-biserial r = −0.163, 95% CI [−0.276, −0.048]. The distribution of ACE categories differed significantly by gender (χ²(3) = 8.76, p = .033, Cramér’s V = 0.152): 27.9% of females versus 19.7% of males reported high ACE burden (≥4 ACEs).

Table 2 presents prevalence rates for individual ACE types by gender with Benjamini-Hochberg FDR correction applied across 10 comparisons (q = .05). After FDR correction, only two ACE types showed significant gender differences: emotional neglect (35.1% female vs. 17.8% male, p_FDR = .003) and household mental illness (29.3% female vs. 16.6% male, p_FDR = .031). Emotional abuse, initially significant in uncorrected analysis (48.2% female vs. 35.7% male, p = .020), did not survive FDR correction (p_FDR = .068).

**Table 2:** Prevalence of individual ACE types by gender (FDR-corrected).

ACE Type	Female (n = 222)	Male (n = 157)	χ²	p (uncorr)	FDR Result
**Emotional Neglect**	78 (35.1%)	28 (17.8%)	12.82	<.001	**✓ SIG**
**Household Mental Illness**	65 (29.3%)	26 (16.6%)	7.47	.006	**✓ SIG**
Emotional Abuse	107 (48.2%)	56 (35.7%)	5.39	.020	NS
Physical Abuse	66 (29.7%)	41 (26.1%)	0.43	.513	NS
Physical Neglect	33 (14.9%)	25 (15.9%)	0.08	.777	NS
Household Substance Abuse	34 (15.3%)	23 (14.6%)	0.03	.862	NS
Domestic Violence	47 (21.2%)	26 (16.6%)	1.19	.275	NS
Parental Separation	66 (29.7%)	41 (26.1%)	0.54	.464	NS
Household Incarceration	17 (7.7%)	16 (10.2%)	0.73	.393	NS
Sexual Abuse	14 (6.3%)	7 (4.5%)	0.57	.451	NS

*Note.* FDR = Benjamini-Hochberg false discovery rate correction at q = .05 across 10 tests. SIG = significant after FDR; NS = not significant after FDR.

### Gender differences in impulsivity

**Fig 1:**
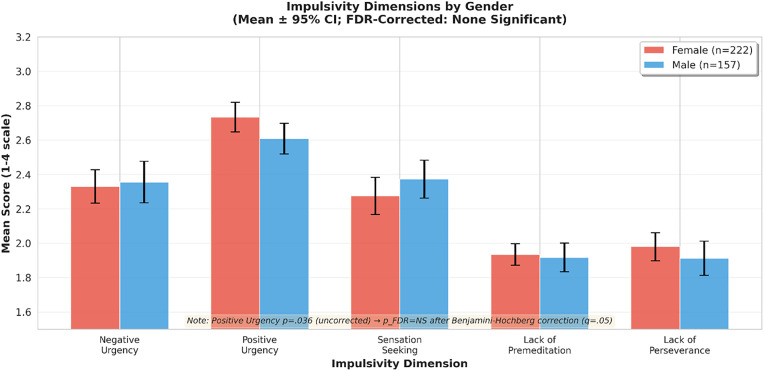
Mean impulsivity scores by gender with 95% confidence intervals. Error bars represent bootstrap 95% CIs (5,000 iterations). After Benjamini-Hochberg FDR correction (q = .05) across five subscales, no gender differences were statistically significant. NS = not significant after FDR.

Table 3 presents mean impulsivity scores by gender. After Benjamini-Hochberg FDR correction across five subscales (q = .05), no dimension showed significant gender differences. Positive Urgency, which appeared significant in uncorrected analysis (p = .036, Cohen’s d = 0.20), did not survive FDR correction. The BH-FDR thresholds for ranks 1–5 were.010,.020,.030,.040, and.050; the smallest p-value (.036) exceeded its threshold (.010), resulting in no significant findings. Fig 1 displays these results graphically with 95% confidence intervals.

**Table 3:** Impulsivity dimensions by gender (FDR-corrected).

Dimension	Female M (SD)	Male M (SD)	Mann-Whitney U	p (uncorr)	FDR Result	Cohen’s d [95% CI]
Negative Urgency	2.33 (0.73)	2.36 (0.77)	17,018.5	.696	**NS**	−0.03 [−0.24, 0.18]
Positive Urgency	2.73 (0.65)	2.61 (0.57)	19,617.0	.036	**NS**	0.20 [0.01, 0.41]
Sensation Seeking	2.27 (0.82)	2.37 (0.71)	15,889.5	.141	**NS**	−0.13 [−0.33, 0.07]
Lack of Premeditation	1.93 (0.48)	1.92 (0.53)	17,942.0	.619	**NS**	0.03 [−0.18, 0.24]
Lack of Perseverance	1.98 (0.62)	1.91 (0.63)	18,477.5	.313	**NS**	0.11 [−0.10, 0.32]

*Note.* FDR = Benjamini-Hochberg false discovery rate correction at q = .05 across 5 tests. NS = not significant after FDR. 95% CIs computed via bootstrap (5,000 iterations).

### ACE-impulsivity associations: Overall and gender-stratified

In the overall sample, ACE total score correlated significantly with four of five impulsivity dimensions: Negative Urgency (ρ = 0.247, 95% CI [0.145, 0.346], p < .001), Positive Urgency (ρ = 0.142, 95% CI [0.042, 0.241], p = .006), Sensation Seeking (ρ = 0.155, 95% CI [0.053, 0.256], p = .003), and Lack of Perseverance (ρ = 0.162, 95% CI [0.059, 0.263], p = .002). Lack of Premeditation showed no significant association (ρ = 0.038, 95% CI [−0.062, 0.140], p = .462).

**Fig 2:**
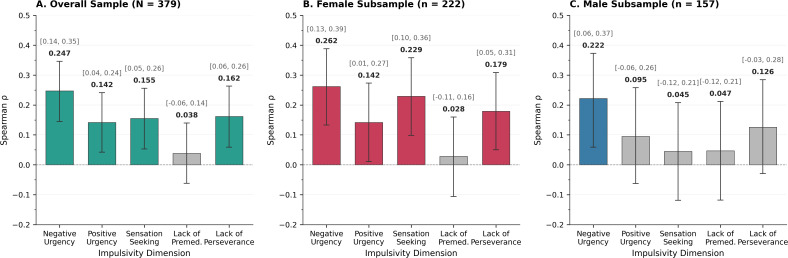
Gender-stratified Spearman correlations between ACE total score and impulsivity dimensions. Panels show (A) overall sample, (B) female subsample, and (C) male subsample. Error bars represent bootstrap 95% confidence intervals (5,000 iterations). Colored bars indicate statistically significant correlations (p < .05); gray bars indicate non-significant correlations.

Gender-stratified analyses revealed divergent patterns in the number of significant associations, though not in correlation magnitudes (Table 4; Fig 2). Among females, ACE total correlated significantly with four dimensions: Negative Urgency (ρ = 0.262, 95% CI [0.133, 0.388], p < .001), Positive Urgency (ρ = 0.142, 95% CI [0.011, 0.273], p = .035), Sensation Seeking (ρ = 0.229, 95% CI [0.098, 0.358], p < .001), and Lack of Perseverance (ρ = 0.179, 95% CI [0.050, 0.309], p = .008). Among males, only Negative Urgency showed a significant association (ρ = 0.222, 95% CI [0.059, 0.373], p = .005).

**Table 4:** Spearman correlations between ACE total and impulsivity dimensions by gender.

Dimension	Overall (N = 379)<br > ρ [95% CI]	Female (n = 222)<br > ρ [95% CI]	Male (n = 157)<br > ρ [95% CI]
Negative Urgency	0.247*** [0.145, 0.346]	0.262*** [0.133, 0.388]	0.222** [0.059, 0.373]
Positive Urgency	0.142** [0.042, 0.241]	0.142* [0.011, 0.273]	0.095 [−0.063, 0.258]
Sensation Seeking	0.155** [0.053, 0.256]	0.229*** [0.098, 0.358]	0.045 [−0.119, 0.208]
Lack of Premeditation	0.038 [−0.062, 0.140]	0.028 [−0.106, 0.160]	0.047 [−0.118, 0.212]
Lack of Perseverance	0.162** [0.059, 0.263]	0.179** [0.050, 0.309]	0.126 [−0.029, 0.285]

*Note.* 95% CIs computed via bootstrap (5,000 iterations). * p < .05. ** p < .01. *** p < .001.

### Cross-gender differences in correlation magnitudes

**Fig 3:**
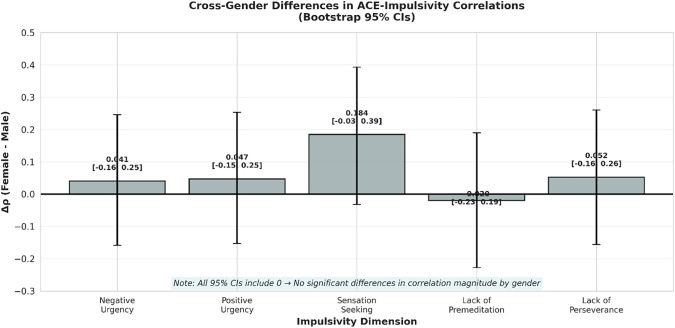
Cross-gender differences in ACE-impulsivity correlations (Δρ = Female – Male) with bootstrap 95% confidence intervals. All confidence intervals include zero, indicating no statistically significant differences in correlation magnitude by gender. The dashed line represents Δρ = 0.

Bootstrap-based tests of cross-gender differences in correlation magnitude (Δρ = ρ_female – ρ_male) revealed no significant differences for any dimension (Fig 3).

Table 5 presents these results. All 95% confidence intervals for Δρ included zero, indicating that observed differences in the pattern of significant associations (more dimensions significant among females) likely reflect greater statistical power in the female subsample driven by higher ACE exposure and variance, rather than fundamentally different per-ACE effects by gender.

**Table 5:** Cross-gender differences in ACE-impulsivity correlations (Bootstrap Method).

Dimension	Female ρ	Male ρ	Δρ (Female – Male)	Bootstrap 95% CI	Includes Zero?
Negative Urgency	0.262	0.222	0.041	[-0.159, 0.246]	Yes (NS)
Positive Urgency	0.142	0.095	0.047	[-0.153, 0.253]	Yes (NS)
Sensation Seeking	0.229	0.045	0.184	[-0.033, 0.393]	Yes (NS)
Lack of Premeditation	0.028	0.047	−0.020	[-0.228, 0.190]	Yes (NS)
Lack of Perseverance	0.179	0.126	0.052	[-0.156, 0.260]	Yes (NS)

*Note.* Bootstrap CIs based on 5,000 iterations. NS = not significant (CI includes zero). The largest difference (Sensation Seeking) approached but did not reach significance.

### Statistical analysis

Analyses used Python 3.12 with SciPy, statsmodels, and pandas libraries. Significance was set at α = .05 with Benjamini-Hochberg false discovery rate (FDR) correction (q = .05) applied within test families [48].

#### Preliminary analyses.

All UPPS-P subscales violated normality (Shapiro-Wilk p < .001), necessitating nonparametric methods. Gender differences in demographics were tested using chi-square tests (Cramér’s V) for categorical variables and Mann-Whitney U tests (rank-biserial r) for ordinal variables.

#### Primary analyses.

*Gender differences in ACE exposure* were examined using Mann-Whitney U tests for ACE Total scores and chi-square tests for individual items, with FDR correction across 10 ACE-type comparisons. Effect sizes included rank-biserial correlation (for Mann-Whitney) and Cramér’s V (for chi-square), with bootstrap 95% confidence intervals (5,000 iterations).

*Gender differences in impulsivity* were tested using Mann-Whitney U tests with Cohen’s d effect sizes and bootstrap 95% CIs, applying FDR correction across five subscales.

*ACE-impulsivity associations* were examined using Spearman correlations separately by gender, with bootstrap 95% CIs (5,000 iterations). Cross-gender differences (Δρ = ρ_female – ρ_male) were computed via bootstrap resampling to avoid parametric assumptions associated with Fisher’s z-transformation [49].

The combined use of gender-stratified analyses and formal moderation testing serves complementary purposes. Stratified correlations characterize the pattern of ACE–impulsivity associations within each gender, identifying which dimensions reach significance for each group. However, differences in patterns of significance between subgroups may reflect differences in statistical power (due to unequal sample sizes: n = 222 females vs. 157 males) rather than genuine moderation. Formal interaction tests in regression models directly assess whether regression slopes differ between genders, providing an explicit test of the differential vulnerability hypothesis while controlling for covariates.

*Moderation analyses* used hierarchical OLS regression predicting each UPPS-P subscale from standardized ACE score, gender (0 = male, 1 = female), and their product term, controlling for age, region, education, employment, and marital status. These covariates were included given significant gender differences in demographics. The interaction coefficient (β_ACE×Gender) and change in R² (ΔR²) quantified moderation effects, with FDR correction applied across five interaction tests.

*ACE-type specificity* was explored using Spearman correlations between individual ACE items and impulsivity dimensions by gender, with familywise FDR correction (q = .05) within each subscale (10 tests per subscale per gender). These analyses were labeled exploratory given high dimensionality.

## Results

### Moderation by gender: Covariate-adjusted models

Hierarchical regression models tested whether gender moderated ACE-impulsivity associations after adjusting for age, region, education, employment status, and marital status (Table 6). Main effects of standardized ACE score were significant for Negative Urgency (β = 0.202, SE = 0.059, p = .001) and Positive Urgency (β = 0.111, SE = 0.050, p = .028), but no Gender × ACE interaction reached statistical significance for any dimension (all interaction p-values > .16). After Benjamini-Hochberg FDR correction across the five interaction tests (q = .05), none remained significant. The change in R² attributable to interaction terms was negligible for all outcomes (ΔR² range: 0.00006 to 0.00467), with the largest interaction observed for Sensation Seeking (β = 0.109, 95% CI [−0.046, 0.264], p = .166, ΔR² = 0.0047).

**Table 6:** Hierarchical regression models testing gender moderation (covariate-adjusted).

Outcome	ACE β (SE)	ACE p	Gender × ACE β	95% CI	Interaction p	ΔR²	FDR
Negative Urgency	0.202 (0.059)	.001	0.012	[−0.140, 0.163]	.880	0.00006	**NS**
Positive Urgency	0.111 (0.050)	.028	−0.028	[−0.156, 0.100]	.669	0.00047	**NS**
Sensation Seeking	0.100 (0.061)	.101	0.109	[−0.046, 0.264]	.166	0.00467	**NS**
Lack of Premeditation	0.004 (0.040)	.928	0.016	[−0.085, 0.118]	.751	0.00025	**NS**
Lack of Perseverance	0.088 (0.050)	.079	0.022	[−0.106, 0.150]	.733	0.00030	**NS**

*Note.* Models control for age, region, education, employment status, and marital status. ACE standardized (M = 0, SD = 1). Gender coded 0 = male, 1 = female. FDR = Benjamini-Hochberg correction at q = .05 across 5 tests. NS = not significant after FDR.

These null moderation findings indicate that while females and males differ in ACE exposure levels, the per-ACE increment in impulsivity does not reliably differ by gender, supporting a differential exposure rather than differential vulnerability interpretation.

### Exploratory analysis: ACE-type specificity

As an exploratory analysis, we examined associations between individual ACE items and impulsivity dimensions separately by gender, applying familywise FDR correction (q = .05) within each subscale (10 tests per subscale). Briefly, among females, FDR-significant associations were observed for multiple ACE types across four dimensions: Negative Urgency showed associations with household substance abuse, emotional abuse, physical abuse, and emotional neglect; Positive Urgency with physical abuse; Sensation Seeking with physical neglect, physical abuse, and emotional neglect; and Lack of Perseverance with domestic violence, emotional abuse, physical abuse, and emotional neglect. Physical abuse emerged as the most consistent predictor across female impulsivity dimensions.

Among males, FDR-significant associations were concentrated exclusively on Negative Urgency and involved three ACE types: emotional abuse, physical abuse, and emotional neglect (strongest association: ρ = 0.341, p < .001). No other dimension showed FDR-significant ACE-type associations in males.

These patterns suggest potential gender-specific ACE-type vulnerabilities, with females showing broader ACE-type impacts across multiple impulsivity facets and males showing focused effects of emotional ACE types on negative urgency. However, given the high dimensionality and exploratory nature of these analyses (100 total comparisons across genders), these findings should be interpreted cautiously and require replication.

## Discussion

This study examined gender differences in adverse childhood experiences and their associations with multidimensional impulsivity in a nationwide Saudi Arabian adult sample. Three primary findings emerged. First, females reported significantly higher ACE exposure than males, with differences concentrated in emotional neglect and household mental illness after rigorous FDR correction. Second, no mean-level gender differences in impulsivity dimensions survived multiple testing correction. Third, and most critically, formal moderation analyses with demographic covariate adjustment revealed no evidence that gender moderates ACE-impulsivity associations. While gender-stratified analyses showed more impulsivity dimensions reaching statistical significance among females (4 of 5) than males (1 of 5), bootstrap-based tests of correlation magnitude differences were uniformly non-significant, and covariate-adjusted Gender × ACE interactions were null across all dimensions. These findings support a **differential exposure** interpretation—males and females differ markedly in ACE burden but show similar per-ACE vulnerability—rather than **differential vulnerability**.

### Differential exposure: Gender differences in ACE prevalence

Our finding that females reported significantly higher total ACE exposure (M = 2.24 vs. 1.75) aligns with some prior research [2] but contrasts with studies showing comparable or higher male ACE prevalence [21]. Critically, FDR-corrected analyses revealed gender differences were specific to emotional neglect (35.1% female vs. 17.8% male) and household mental illness (29.3% female vs. 16.6% male), both reflecting psychological and emotional adversity rather than physical maltreatment. This pattern may reflect unique features of Saudi family structure and gender socialization.

In patriarchal societies with clearly delineated gender roles, females may experience greater psychological control, restricted autonomy, and emotional invalidation, elevating risk for emotional neglect [43,45]. In Saudi Arabia’s traditional family system, several concrete pathways may link societal-level gender norms to individual-level experiences of emotional neglect. First, cultural norms may prioritize sons’ emotional needs and accomplishments while normalizing emotional restraint toward daughters, such that girls’ emotional expressions receive less validation and fewer responsive reactions from caregivers. Second, the guardianship system historically limited women’s independent decision-making in education, employment, travel, and marriage; while formal restrictions have eased considerably since the 2019 reforms, family-level practices often lag behind legal change, and young women growing up under such restrictions may retrospectively interpret constrained autonomy as a form of care deficit. Third, family honor norms (sharaf) place disproportionate behavioral expectations on daughters, where emotional expression or disclosure of distress may be discouraged as bringing shame, leading to a subjective experience of emotional needs being unmet. Saudi cultural norms traditionally emphasize female obedience, modesty, and family honor, potentially creating environments where emotional needs are subordinated to family reputation [43,45,50]. The significant elevation in household mental illness reports among females may similarly reflect gendered exposure to caregiving burdens within the household, as women traditionally bear primary responsibility for family caregiving and may therefore have greater awareness of family members’ psychological difficulties [51].

Notably, we found no significant gender differences in physical or sexual abuse after FDR correction, contrasting with Western meta-analyses showing higher female sexual abuse prevalence [23]. This may indicate genuine cultural differences in abuse patterns, though substantial underreporting of sexual abuse—particularly among males due to stigma—cannot be ruled out [52]. Cultural taboos surrounding discussion of sexual topics may suppress reporting rates for both genders in Saudi contexts.

### No reliable gender differences in impulsivity

Contrary to Western meta-analytic findings showing higher male sensation seeking and higher female urgency [31,32], we found no significant gender differences in any impulsivity dimension after FDR correction. The apparent female elevation in Positive Urgency (uncorrected p = .036, d = 0.20) did not survive multiple testing correction, highlighting the importance of rigorous statistical control in high-dimensional research.

This divergence from Western patterns may reflect cultural influences on impulsivity expression and reporting. In collectivistic societies emphasizing emotional restraint and conformity, self-reported impulsivity may be influenced by social desirability biases, potentially suppressing gender differences observed in individualistic Western contexts [36,50]. Saudi cultural norms particularly emphasize female behavioral restraint and male emotional stoicism, which may influence how each gender perceives and reports impulsive tendencies [45]. Alternatively, the null finding may reflect genuine cultural parity in trait impulsivity, challenging assumptions about universality of gender differences in personality traits.

The relatively low reliability of Lack of Premeditation (α = .55) in our sample warrants consideration. While this subscale showed no gender differences or ACE associations, the poor internal consistency limits confidence in these null findings. Future research should employ longer impulsivity measures or culturally adapted items to ensure adequate reliability across all dimensions.

### Differential exposure, not differential vulnerability

The central contribution of this study lies in rigorously distinguishing between differential exposure and differential vulnerability hypotheses. Superficially, our gender-stratified analyses appeared to suggest differential vulnerability: ACEs correlated significantly with four impulsivity dimensions in females but only one in males. However, three lines of evidence refute this interpretation.

First, bootstrap-based tests of cross-gender correlation magnitude differences were uniformly non-significant; all Δρ confidence intervals included zero. The largest difference (Sensation Seeking: Δρ = 0.184) approached but did not reach significance, suggesting females may show somewhat stronger ACE-sensation seeking associations, but this requires replication. Critically, the pattern of “more significant associations in females” likely reflects greater statistical power driven by females’ higher ACE exposure and variance rather than fundamentally different per-ACE effects.

Second, formal moderation analyses testing Gender × ACE interactions—the appropriate statistical framework for differential vulnerability—were consistently null across all dimensions after covariate adjustment (all p > .16, all ΔR² < 0.005). These analyses explicitly model whether ACE slopes differ by gender, directly testing the differential vulnerability hypothesis. The null interactions remained non-significant after FDR correction and inclusion of demographic covariates (age, region, education, employment, marital status) that differed by gender, ruling out confounding as an explanation.

Third, the largest interaction (Sensation Seeking: β = 0.109, ΔR² = 0.0047) explained less than half of one percent of variance, well below thresholds for practical significance. Thus, even if a small moderation effect exists, its magnitude is clinically negligible.

These convergent findings support the differential exposure framework articulated by Rutter (2006) and Kessler et al. (2010) [4,40]: males and females show similar vulnerability to ACEs but differ in exposure patterns. This interpretation aligns with research showing comparable ACE effects across genders for most psychiatric outcomes [25,29] and challenges assumptions about female-specific trauma vulnerability that pervade clinical discourse.

### ACE-type specificity: Gender-differentiated pathways

Exploratory FDR-corrected analyses suggested potential gender differences in which ACE types associate with which impulsivity dimensions. Among females, physical abuse emerged as the most consistent predictor, showing FDR-significant associations with Negative Urgency, Positive Urgency, Sensation Seeking, and Lack of Perseverance. Among males, associations were concentrated on Negative Urgency and involved emotional abuse, physical abuse, and particularly emotional neglect (strongest: ρ = 0.341).

This pattern is consistent with the possibility that females may show broader, more diffuse associations between physical maltreatment and regulatory domains, whereas males may show more focused associations between emotional adversity and emotion-driven impulsivity. These exploratory findings align with research suggesting that physical abuse may be associated with alterations in multiple self-regulatory systems through chronic stress activation, though the mechanisms remain to be established in prospective research [16,17,20]. In contrast, the concentration of male associations on Negative Urgency is consistent with theoretical models suggesting that emotional deprivation may be particularly linked to deficits in emotion-driven impulse control [10,19], though the cross-sectional nature of our data precludes causal inference.

However, these ACE-type findings are exploratory, derived from 100 comparisons across genders, and require replication. Future research should use confirmatory approaches (e.g., preregistered hypotheses, elastic net regression) to identify robust ACE-type-specific effects while controlling family-wise error.

### Cultural context and implications for middle Eastern research

This study represents the first examination of gender differences in ACE-impulsivity pathways in a Middle Eastern population using multidimensional impulsivity frameworks and rigorous statistical controls. Several culturally specific considerations warrant discussion.

First, the concentration of female ACE elevation in emotional domains (neglect, household mental illness) rather than physical abuse aligns with qualitative research on Arab family dynamics, where psychological control and emotional invalidation may be particularly salient for females navigating restrictive gender role expectations [43,51]. Saudi Vision 2030 reforms have begun addressing female autonomy and social participation [53], but deeply entrenched patriarchal family structures may take generations to transform.

Second, the absence of gender differences in impulsivity challenges Western-derived assumptions about universality of personality trait distributions. Cross-cultural psychology increasingly recognizes that personality structure and expression vary across cultural contexts [54], with collectivistic societies potentially showing different patterns than individualistic Western nations. Our findings suggest gender differences in impulsivity observed in WEIRD samples (Western, Educated, Industrialized, Rich, Democratic) [35] may not generalize to Middle Eastern contexts.

Third, potential underreporting of stigmatized ACEs (particularly sexual abuse, substance abuse) in Saudi samples limits comparability to Western prevalence estimates. Cultural taboos surrounding discussion of family problems, sexuality, and mental health may suppress disclosure rates, potentially attenuating observed ACE-outcome associations. Future research employing anonymous reporting, behavioral measures, or informant reports may yield different estimates.

Fourth, the role of Islam and religious norms warrants consideration in interpreting both the low overall impulsivity scores and the patterns of ACE disclosure in this Saudi sample. Islamic teachings explicitly value patience (sabr), deliberation, and self-restraint, and discourage impulsive behavior across multiple domains relevant to the UPPS-P framework. The five daily prayers (salat) impose temporal structure and routine self-regulation that may serve as behavioral scaffolding against impulsive action. Expected abstinence from alcohol, gambling, extramarital sexual activity, and substance use—behaviors that feature prominently in Sensation Seeking and Positive Urgency items—may suppress endorsement of these items through genuine behavioral restraint, internalized religious values, or social desirability in reporting. Additionally, religious framing of adversity (e.g., viewing suffering as a test of faith) and the Islamic emphasis on family privacy (satr) may reduce disclosure of certain adverse childhood experiences, particularly those involving stigmatized behaviors such as household substance abuse, domestic violence, or parental mental illness. The absence of a direct religiosity measure in this study prevents us from disentangling the relative contributions of religious belief, practice, and broader cultural norms to the observed patterns, and represents an important direction for future research.

### Clinical and policy implications

Our findings carry important implications for trauma-informed intervention in Saudi Arabia and potentially other Middle Eastern contexts. First, the differential exposure pattern suggests prevention efforts should target gender-specific ACE types: emotional neglect and household mental illness for females, and potentially different targets for males. Family-based interventions addressing emotional validation, autonomy support, and mental health stigma may be particularly beneficial for at-risk females.

Second, the absence of differential vulnerability suggests trauma-focused interventions need not be fundamentally different for males versus females. While presentation may vary (e.g., ACE-type exposure, symptom profiles), the basic per-ACE impact on regulatory capacities appears comparable. This supports universal trauma-informed care principles adapted for ACE-type specificity rather than separate treatment tracks by gender [55].

Third, the prominence of emotional neglect in Saudi female ACE profiles highlights need for culturally adapted parenting interventions. Western parenting programs emphasizing warmth, validation, and autonomy support may require modification to align with collectivistic values while still addressing emotional needs [51].

Fourth, screening for ACE exposure in clinical and public health settings should target both genders. The focus on female trauma vulnerability may lead to under-identification of male ACE exposure and associated mental health needs, perpetuating gender disparities in service access.

### Strengths and limitations

Strengths include nationwide sampling across all Saudi regions, validated Arabic measures, zero missing data on primary variables, multidimensional impulsivity assessment, and rigorous statistical methods including FDR correction for multiple testing, bootstrap confidence intervals, and covariate-adjusted moderation models. This study represents the most methodologically rigorous examination of gender differences in ACE-impulsivity pathways in Middle Eastern populations to date.

Several limitations warrant consideration. First, the cross-sectional design precludes causal inference. While we framed ACEs as predictors of impulsivity based on temporal precedence and theoretical models [16], bidirectional or third-variable explanations cannot be ruled out. Longitudinal designs tracking ACE exposure and impulsivity trajectories across development are needed.

Second, convenience sampling with overrepresentation of educated, urban, Central Region residents limits generalizability. Our sample likely underrepresents lower-SES adults, rural populations, and Northern/Southern regions. Socioeconomic gradients in ACE exposure are well-established [25]; our estimates may underestimate true population prevalence.

Third, retrospective self-report of childhood experiences introduces recall bias and potential for mood-congruent memory distortion [56]. Prospective longitudinal designs or multi-informant approaches would strengthen causal inference, though retrospective ACE assessment remains standard in the field given pragmatic constraints.

Fourth, the below-threshold reliability of Lack of Premeditation (α = .55) limits confidence in null findings for this subscale. While consistent with prior short-form research in non-Western samples [47], future studies should use longer measures or culturally adapted items.

Fifth, we did not test measurement invariance of the UPPS-P across gender, though null moderation findings reduce concern about biased comparisons. Future research should conduct multi-group confirmatory factor analysis to establish configural, metric, and scalar invariance before comparing means or associations across genders.

Sixth, cultural factors may have suppressed reporting of stigmatized ACEs (sexual abuse, substance abuse) or influenced impulsivity self-reports through social desirability. Anonymous behavioral measures or informant reports would complement self-report data.

Seventh, we did not include a measure of religiosity or religious practice, despite the central role of Islam in Saudi daily life. Religious commitment may independently influence both impulsivity expression (through internalized values of patience and self-restraint) and ACE disclosure (through norms of family privacy and acceptance of suffering). Future studies should incorporate validated religiosity scales to examine whether religious practice moderates ACE–impulsivity associations or influences reporting patterns.

### Future directions

Several research directions emerge from this work. First, longitudinal studies tracking ACE exposure and impulsivity development from childhood through adulthood in Middle Eastern samples would clarify causal pathways and identify sensitive periods for intervention. Second, examination of potential mechanisms linking ACEs to impulsivity—including emotion regulation, executive function, stress physiology, and attachment—would illuminate targets for treatment. Third, expanded ACE assessments incorporating culture-specific adversities (e.g., honor-based violence, forced marriage, war exposure) may provide more comprehensive adversity profiles. Fourth, investigation of resilience factors protecting against ACE sequelae in Middle Eastern contexts (e.g., religiosity, family cohesion, cultural values) could inform strengths-based interventions. Fifth, replication in other Middle Eastern countries and diverse SES samples would establish generalizability. Finally, intervention research testing culturally adapted trauma-focused treatments in Saudi and regional samples is critically needed.

## Conclusion

This study demonstrates that gender differences in ACE-impulsivity associations among Saudi adults reflect differential exposure—females experience more adverse childhood experiences, particularly emotional neglect—rather than differential vulnerability. While more impulsivity dimensions showed significant ACE associations among females, rigorous statistical tests revealed no significant differences in correlation magnitude, and formal moderation analyses with demographic covariate adjustment yielded consistently null Gender × ACE interactions. These findings challenge assumptions about female-specific trauma vulnerability and suggest universal trauma-informed approaches with ACE-type-specific modifications may be optimal. The concentration of female ACE elevation in emotional domains highlights need for culturally informed prevention addressing psychological maltreatment in patriarchal family contexts. Future research employing longitudinal designs, behavioral measures, and culturally expanded ACE assessments will further illuminate gender-differentiated pathways from childhood adversity to self-regulatory outcomes in Middle Eastern populations.
